# A qualitative study to explore communication skills in veterinary medical education

**DOI:** 10.5116/ijme.542a.975d

**Published:** 2014-10-11

**Authors:** Wendy J. Hamood, Anna Chur-Hansen, Michelle L. McArthur

**Affiliations:** 1 School of Animal and Veterinary Science, University of Adelaide, Roseworthy Campus, Roseworthy, South Australia; 2 School of Psychology, University of Adelaide, South Australia

**Keywords:** Communication skills, veterinary education, practitioner perspective, qualitative research

## Abstract

**Objectives:**

To explore and gain an understanding of what “clinical communication skills” mean to veterinarians working in private practice and what implications this might have for veterinary medical education.

**Methods:**

Qualitative research methods were used to purposefully sample a range of veterinary practitioners from a pool of South Australian veterinary practices who were interviewed to determine their understanding of what communication skills mean in the context of veterinary practice. Interviews were conducted with fourteen veterinary practitioners. Participants were sampled from a range of ages, veterinary schools of graduation plus urban and rural locations. Interview transcripts were analysed for themes, definitions and contexts.

**Results:**

Participants’ accounts included a number of skills which they considered to be “communication”. Some of the definitions of these skills parallel communication skills and competencies for human medicine on which communication skills training incorporated into veterinary curricula to date have largely been based. However, the veterinarians in this study also raised interesting contextual differences unique to the veterinary profession, such as communication with the animal, selling service, discussing money in relation to decisions for care, and communicating about euthanasia.

**Conclusions:**

Veterinary practitioners require high level communication skills. Education and training in veterinary medicine may be better tailored to reflect the unique context of the veterinary profession.

## Introduction

The recognition that communication skills are as important in veterinary medicine as they are in human medicine, and therefore must be taught in veterinary curricula, is a relatively new concept and research is limited.^1^ By contrast, there is a substantial body of literature in human medicine spanning several decades.^2^ In human medicine myriad studies consider the definition of communication skills and competencies, how they can be translated into clinical practice, what impact they have on clinical outcomes, and how they can be best incorporated into undergraduate, postgraduate and professional training, including continuing professional development.

Some scholars, notably Jane Shaw^3^, Cindy Adams and colleagues^4^, ^5^ have applied human medicine communication skills research and training methods to veterinarians and veterinary students, highlighting the parallels and similarities between the two professions. Both are service providers and health care professionals who work to improve patient health. In both professions success and satisfaction are dependent on interactions with humans. Shaw and colleagues^6^ proposed that the structure and content of interviews with patients/clients is similar, and thus the measures used to assess and evaluate medical interactions can also apply to veterinarians.

Parallels have been observed not only in skills, but also in their delivery. For example, Shaw and colleagues^7^ have demonstrated that women veterinarians are more relationship focussed during consultations, with better skills in establishing and building rapport, as compared with men. This gender difference replicates findings in human medicine.^7^ In a focus group study by Coe et al,.^8^ communication skills competencies needed when working with clients and companion animals mirrored that of human medicine: educating clients; providing choices; use of two way communication between the practitioner and the client; breakdowns in communication that impair the interaction; and challenges in communication such as discussion of finances, client misinformation, more than one client in the interaction and time constraints. It is also clear that as with human medicine, where there are breakdowns in communication, the relationship is adversely affected.^8^

However, veterinary medicine is not the same as human medicine. The veterinary consult can be described as tripartite, involving the owner, patient and the veterinarian.^9^ Shaw and colleagues 3 acknowledge that whilst there are similarities there are also contextual differences. The most obvious, of course, is that the patient is an animal. Radford and colleagues^9^ included the need to attend to the animal’s comfort, establishing the role and purpose of the animal to the owner and using the animal to build a relationship with the client. Shaw et al.^3^ also list aspects of the human-animal bond, which might be comparable to, but are not the same as the parent-child bond; euthanasia and the challenges that brings for both the client and also the veterinarian; the death of patients, which is far more frequent for veterinarians than most medical practitioners (dependent on specialty); and financial considerations which might result in the withholding of treatment or the euthanasia of an animal, situations that do not occur as a matter of course in human medicine, particularly in countries like Australia. Of relevance too, is the fact that whilst many doctors run their practice as a business, many work in public hospital settings and have access to government funded health schemes for payment to which veterinarians do not have access. Indeed, research has been conducted that specifically considers the monetary aspects of veterinary care^10^ with stronger communication competency related to higher financial returns.^11^

The veterinarian-animal-client situation is considered to be comparable to the paediatrician-child-parent situation.^3^, ^12^ Paediatrics, it has been argued, requires communication that rarely involves a dyad^13^ and is a specialty that is unique and distinct from other areas of human medicine.^14^ Paediatricians usually work within a triad (doctor-child-parent), other family members may also be involved, the age of the child impacts communication (for example preverbal as compared with the child able to use and understand language), family dynamics affecting the interaction, including decision making, plus parents and children having different needs in the consultation. Parents value physicians who try to understand their perspectives, pay adequate attention and respect to their concerns about the child, and who build a partnership in which the child’s feelings and the parents are taken into consideration.^14^ Ways in which the child can be compared with the animal in veterinary practice are obvious. This is especially so given that in contemporary Western society companion animals are often-times viewed as members of the family,.^12^ and specifically children.^15^

Whilst it may be true that some of the communication skills needed in veterinary medicine are the same as those needed in human medicine, there is little research that specifically considers these skills in relation to the unique contexts commonly encountered in veterinary practice. This study aims to explore communication skills in the context of veterinary practice and implications for veterinary medical education.

## Methods

### Study design

Qualitative research methods were used for this study. Individual interviews were chosen in preference to focus groups due to practical limitations of participants being able to attend focus groups at a given time.

### Participants

The University of Adelaide School of Animal and Veterinary Sciences has a pool of veterinary practices within the state of South Australia that provide clinical placements for veterinary students in their clinical years of training. At the time of this study, approximately one hundred local practices offered placements. Purposeful sampling was used to ensure a range of practices from urban and rural locations were targeted from within this pool of practices. Thirty five letters were sent in hard copy and via email, asking practice owners if they or any of their associates would like to participate in a study on communication skills in veterinary training. Sixteen veterinarians indicated they were willing to participate in the study and of these fourteen were interviewed. Five of the participants were women. Age and year of graduation of participants was relatively evenly distributed, ranging between 26 to 62 years of age, and 2010 to 1972 for year of graduation. Three participants were veterinary associates while the remainder were veterinary practice owners, with eight veterinarians from urban practices, five rural and one semi-rural. Of the veterinarians interviewed, there were three graduates each from the University of Melbourne, the University of Sydney and Murdoch University, two from the University of Queensland and three from veterinary schools in the United Kingdom and Netherlands.

### Procedure

The first and third authors conducted interviews, the former a veterinary surgeon with 20 years’ experience working in local South Australian practices, the latter a veterinary educator specialising in teaching communication skills. Four of the rural veterinarians were interviewed over the phone and one face to face at Roseworthy Campus; all other interviews were conducted face to face, at the place of the veterinarian’s practice. Following each interview, demographic information was collected on age, year of graduation, practice position (owner or associate), and experience in veterinary practice and with supervising veterinary students.

Interviews were semi-structured, with some set question prompts. Specifically, potential participants were advised that the study involved seeking their thoughts on what they understand about “clinical communication skills”, how useful they consider such skills and how their understanding of communication skills might be different to veterinary students. Participants were free to elaborate and introduce any other information that they felt was relevant. Prompts that were asked included: “What do you think are the most important aspects of communicating with clients in a consult?”; “What is the value of teaching clinical communication skills to veterinary students?” and “What do you understand about communication skills compared to students?” Interviews lasted approximately 45 min (range 38min to 1 hour 20min). All interviews were audiotaped and transcribed verbatim. Data saturation was evident after the fourteenth participant’s interview, meaning, no new information was apparent.

### Data analysis

The data were analysed thematically, following the methodology as outlined by Braun and Clarke.^16^, ^17^ The first author immersed herself in the data, becoming familiar with the material within it. The data were examined in relation to the research question “What does the term ‘communication skills’ mean to veterinary surgeons in the context of veterinary practice?”, and initial codes established. These were refined into themes, and then further collapsed until a set of definitions was produced from the data, with corresponding exemplars from interviews. The final themes, definitions and contexts were examined by the second and third authors, who crosschecked the results back to the raw data. These steps in analysis fulfil the expectations of trustworthiness and rigour as outlined by Tracy.^18^

### Ethical consent

The University of Adelaide Human Research Ethics Committee approved the project.

## Results

All participants discussed at length the meaning of “communication skills” in as they relate to veterinary practice. All participants believed that good communication skills are an essential attribute for veterinarians and all were able to articulate what “communication skills” are in practice. Some of their definitions parallel the conceptualisation of communications skills and competencies as they are viewed in human medicine. The skills articulated by participants, included communication skills relating to; opening the discussion, building the relationship, gathering information, explanation and planning, structuring the consult and closing the session and tailoring the communication specifically to the client’s needs. However, veterinarians raised four interesting contextual differences as described below.

### Communication with the animal

Participants explained that an important communication skill in veterinary medicine is to make the animal relaxed. A genuine interest in the animal was deemed important in being able to achieve this, including addressing the animal by name, if the animal has one. A core skill that was articulated was the ability to balance communication with the animal with communication with the human client, rather than simply focussing on one or the other. Participants described drawing upon different sensory modalities to communicate with the animal: looking, listening, smelling, touching, and hearing, and that communication with the animal commences as soon as the veterinarian enters the waiting room.

“Also communicating that you are interested in the pet as well, you have to communicate with the animal as well as the owner.” (Interview 7, 35 years, female)

### Selling service

Participants considered that communication skills are important in relation to providing service. It was argued that veterinary practice is a service industry: you need to win customers and make sure they come back. By communicating standards of care-for example, by covering all aspects of preventative care within time constraints, participants viewed communication skills as essential for attracting and keeping clients, who were viewed as customers. Catering for the client’s emotional needs and personality type was considered to part of service delivery. One communication strategy articulated by participants was “having a spiel” for routine things, for example, selling heartworm prevention in a vaccination consult. A challenge for practitioners, participants explained, is being skilled in communication so that the emotional side of pet ownership can be respected alongside providing service to the client, but also considering the commercial aspects of running a business.

“Customer service and client relations are the number one thing with vet practice, because it doesn’t matter how good a vet you are, if you can’t relate to people they are not going to come back or they are not going to understand what you have done. It’s really important in a business. We are an industry of customer service whether we like it or not.” (Interview 10, 37 years, female)

### Communicating about money, decisions and related costs

Related to selling services was explaining costs. Participants viewed giving clients enough information to make reasonable decision as an important skill. When discussing cost it was considered important to be flexible, not to make assumptions and approach the topic with an open mind. Comprehensible information provision was seen to be a communication skill. Participants discussed the need to give information in a manner that did not cause offence, anger or distress to the client, and in a way that encourages the owner to want to return again as a repeat client. Foremost in participants’ discussions were negotiations and information provision about money. Participants explained that discussing costs of care could be associated with confrontation and complaints. They considered important skills for veterinarians to include knowing when to be compassionate and when to be stern when clients argued about fees and charges, particularly when this may compromise the welfare of the animal. Empathy was seen to be an essential communication skill when talking with clients about cost. Making sure that costs are not unexpected, and clients are aware that some treatments can be cost prohibitive, particularly in relation to costs and benefits to the animal, were also discussed. The need to understand that financial constraints often affected the ability of veterinary practitioners to provide animals with the best standard of care was also mentioned. Related to the discussion of costs, participants described the importance of behaving in an ethical manner – for example, not over-servicing clients.

“Because reality is that going to the vet is expensive, so it’s then that you communicate, to have clients comfortable about the large expense that they are spending.” (Interview 11, 44 years, male)

### Communicating about euthanasia

Participants spoke about the importance of good communication skills when speaking to clients about euthanasia. The age of the client, the meaning of the animal to the client, and the clients’ religious and cultural backgrounds were factors that participants considered when talking about ending an animal’s life. Core skills noted by participants included explaining in a way that is appropriate for the client in relation to such factors. Participants considered it important to take time to explain the reasons for euthanasia as well as the process. Explanations to children were viewed as requiring specific skills, but for all clients, participants felt that they may cause distress if the appropriate terminology is not used. In providing information to clients, participants felt that for some, dealing with decision-making and emotion is hard, and certain clients may not understand what the veterinarian is communicating, due to their heightened emotional state. Participants felt that they needed good communication skills in order to recognise when discussions about euthanasia might be wanted, as number of clients may find it difficult to bring up the topic.

“We had a situation where a mother had her young child with her when the dog was euthanized. The vet described it to the child as ‘We are going to put Buddy to sleep.’ You know as we do, that is a fairly common expression in the veterinary world. The mother actually brought the child back the next day and said, ‘Look can you please explain to him that Buddy was euthanized, not put to sleep.’ They now have a child who is terrified of going to bed, and that he’s not going to wake up in the morning.” (Interview 14, 33 years, female)

## Discussion

This study considers veterinarians’ perceptions about specific contexts relevant to the veterinarian-animal-client encounter. Whilst it is clear that parallels between human and veterinary medicine exist with regards to communication skills, this study highlights the unique contextual differences that are evident when working with animals. Veterinarians are required to relate to people and animals and it is this unique interaction that characterises the profession. Whilst generic communication skills were discussed by participants in the context of veterinary medicine, arguably there were four contextual differences only relevant to veterinarian practice, and not to human medicine: communication with the animal; selling service; communicating about money, decisions and related costs; and communicating about euthanasia.

Communicating with animals is an obvious skill required of veterinarians. Interestingly though, the communication research to date in veterinary medicine has not adequately examined communication from veterinarian to animal. Studies utilising the Roter Interaction Analysis System have analysed the triadic nature of veterinary medicine small animal consultations.^6^, ^19^-^21^ However, there was little focus on the type, amount and impact of communicating with the animal in terms of clinical outcomes. Various studies have reported clients’ satisfaction with veterinarians who interact positively with their animal^20^, ^22^ but further research should examine the impact on the animal and the veterinarian. There is no doubt that information is communicated between the animal and the veterinarian during the consultation. This aspect of communication is a fruitful area for further empirical study. The animal will use nonverbal means of communication and an understanding of these is essential for the effective veterinarian. Furthermore, communicating with the animal is also important in terms of safety and being aware of animal cues and behaviours that indicate approach and avoidance. Appreciating the nonverbal cues of patients in the veterinary context is a communication skill that requires further research.

Selling service, it can be argued, is unique in veterinary medicine as compared to other health care professions where promotion of services is also relevant, given that an appraisal of cost by the animal’s owner may result in the death of the animal. Being able to sell services is of vital importance, not only for the potential welfare of the animal, but also to individuals within the veterinary profession, in the context of an oversupply of new graduates.^23^, ^24^ Previous research suggests that veterinarians may be underservicing clients as a result of misjudging clients’ interest or a lack of exploration of clients’ needs.^22^, ^25^, ^26^ A study conducted by Baguley^23^ has predicted low growth in demand for companion animal veterinary services in Australia over the next decade due to a forecasted low growth or possible decline in pet ownership. In order for practices to improve revenue in companion animal practice an increase in average client fees will be needed.^23^ The changing role of the production animal veterinarian from traditional practitioner to that of consultant requires veterinarians to be able to persuade producers to invest in an expanding range of services that veterinarians can provide.^27^ Consequently, communication skills programs may need to consider approaches in selling service to clients an integral component of their curriculum. The ethical considerations around the use of communication to sell services are also a matter for the education, training and continuing professional development of veterinarians.^28^

Related to selling services is the need to have discussions of cost, a fundamental conversation noted by veterinarians in this study. These discussions have been reported to be one of the most concerning to recent graduates^29^ and more established veterinarians.^30^ There is limited information pertaining to discussions of costs in veterinary medicine but it is acknowledged there are significant differences between clients and veterinarians in their beliefs as to the important factors to consider in such conversations. For example, veterinarians tend to explain costs in terms of services rendered and time,.^30^ while clients want outcomes related to their animal’s health and welfare.^10^ The dearth of literature in this area is surprising given the emotional climate in which these conversations may occur and coupled with nearly 30% of consultations likely to include a discussion of cost.30 A model such as SPIKES,.^31^ a mnemonic where six communication skills are incorporated into a protocol to direct clinicians in breaking bad news, could be developed to serve as a guide for navigating discussions about costs. This is worthy of further research. Such a model needs to be tested for applicability but ultimately would help educators in guiding veterinarians to optimally manage these challenging conversations.

Veterinarians are required to explicitly discuss euthanasia. Euthanasia in veterinary medicine is a core and profound responsibility and its explicit discussion with clients is unique. End of life decision-making requires the intentional use of many skills including empathy, nonverbal communication, building rapport, listening, using client friendly language, honesty, and understanding the client’s life circumstance.^32^ However, many veterinarians feel their veterinary training ill-prepared them to manage these discussions effectively.^33^ Furthermore, veterinarians may show reluctance in initiating a discussion of euthanasia and this may be in part attributed to limited formal training.^34^ It is very likely that veterinarians, as well as clients find this a difficult conversation.^34^ Given that each veterinarian will conduct, on average, nearly eight euthanasia procedures per month,.^33^ along with reports that one third of clients talk to their vets about the loss of their animals,.^35^ highlights the need for effective communication skills in this domain. Approximately 75% of a sample of North American veterinarians believed that there should be a stronger emphasis on communication skills training for terminally ill patients and their owners.^36^

In Australia, the United States, and in many parts of Europe, as well as in some parts of Asia, companion animals are now viewed as family members.^37^ Farmers may grieve individual or multiple stock losses for a variety of reasons both financial and emotional, including the loss of generational heritage.^37^ Demands on veterinarians to master communication skills have never been so great, in light of the challenges faced by the profession, as raised by Gardiner.^38^ Poor communication skills not only have the potential to threaten job satisfaction, professional and personal success as well as the clinical outcomes of encounters,.^19^ but may lead to litigation^39^, ^40^ and distress for the client. In some instances, this distress could be quite significant. The ability to recognise normal human emotional reactions, and assess where there is a need for referral to another professional, for example, a general practitioner or psychologist, cannot be considered an optional skill. It is important that veterinarians receive communication skills training that will prepare them for the demands of the profession. This study contributes to this need by identifying the skills that veterinarians have recognised as relevant to their profession, and through identification of those that should be incorporated into undergraduate and postgraduate curricula and continuing professional development.
